# Discovery and cultivation of prokaryotic taxa in the age of metagenomics and artificial intelligence

**DOI:** 10.1093/ismejo/wrag012

**Published:** 2026-01-30

**Authors:** Diego Javier Jiménez, Ramona Marasco, Júnia Schultz, Carlos Andrés Díaz Rodríguez, Juan Nogales, Luis Miguel Rodriguez-R, Jörg Overmann, Alexandre Soares Rosado

**Affiliations:** Biological and Environmental Sciences and Engineering Division (BESE), King Abdullah University of Science and Technology (KAUST), 23955-6900, Thuwal, Kingdom of Saudi Arabia; Biological and Environmental Sciences and Engineering Division (BESE), King Abdullah University of Science and Technology (KAUST), 23955-6900, Thuwal, Kingdom of Saudi Arabia; Biological and Environmental Sciences and Engineering Division (BESE), King Abdullah University of Science and Technology (KAUST), 23955-6900, Thuwal, Kingdom of Saudi Arabia; Católica Biomedical Research Centre, Medicine Faculty, Universidade Católica Portuguesa, 2780-156, Oeiras, Portugal; Gulbenkian Institute for Molecular Medicine, 2780-156, Oeiras, Portugal; Department of Systems Biology, Centro Nacional de Biotecnología, CSIC, C/Darwin nº 3. 28048, Madrid, Spain; Department of Chemistry and Bioscience, Aalborg University, Fredrik Bajers Vej 7H9220, Aalborg, Denmark; Leibniz Institute DSMZ-German Collection of Microorganisms and Cell Cultures, and Institute for Microbiology, Faculty of Life Sciences, Technical University of Braunschweig, 38124, Braunschweig, Germany; Current affiliation: Bavarian State Collections of Natural History, Munich, Germany; and Faculty of Biology, Ludwig-Maximilians-Universität München, 80638, Munich, Germany; Biological and Environmental Sciences and Engineering Division (BESE), King Abdullah University of Science and Technology (KAUST), 23955-6900, Thuwal, Kingdom of Saudi Arabia

**Keywords:** culturomics, isolation, genotype–phenotype inference, genome-scale metabolic models, machine learning, metagenome-assembled genomes, microbiome perturbation

## Abstract

Despite advances in sequencing, microbial genomics, and cultivation techniques, the vast majority of prokaryotic species remain uncultured, which is a persistent bottleneck in microbiology and microbial ecology. This perspective outlines a conceptual framework to improve the transition from genome-resolved metagenomics to the targeted isolation of yet-uncultured prokaryotic taxa. The proposed framework integrates the induced reshaping of microbiomes, genome-based inferences of physiological and phenotypic traits, culture media design, and targeted culturomics, enabling hypothesis-driven cultivation. In addition, this manuscript addresses the critical limitations in the field, including the sequence-to-function gap, and emphasizes the synergistic potential of experimental microbiology, microbial ecology, metagenomics, and artificial intelligence–based predictions to enhance rational and actionable roadmaps for discovering and cultivating novel prokaryotic lineages.

## Graphical Abstract

**Graphical Abstract:**
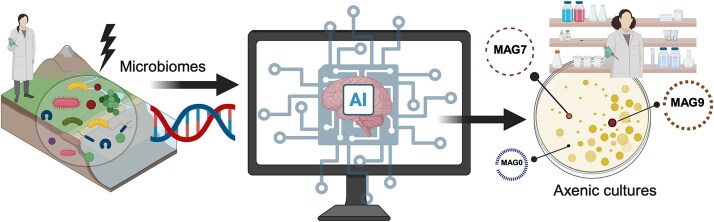
BioRender.com was employed to create the figure

## Introduction

Advances in genome-resolved metagenomics (see Table 1 for a glossary) has improved the systematics of prokaryotes and expanded the discovery of novel taxa across a wide range of ecosystems [1–4]. However, our understanding of the functions of newly identified genomes and how to isolate the corresponding microorganisms is still limited. The exact fraction of prokaryotic diversity belonging to yet-uncultured species remains debated [5–7], as does the estimation of the total microbial diversity on this planet [8, 9]. It is widely recognized that far more than 90% of known prokaryotic species have never been brought into culture [4, 10]. Even among species for which genome sequences are available, recent estimates suggest that >75% remain uncultivated [11, 12]. In fact, from the trillions of predicted prokaryotic species inhabiting Earth [13, 14], currently <22 000 have been formally described (https://lpsn.dsmz.de/text/numbers) of which ~20 000 have been deposited as pure cultures in the German Collection of Microorganisms and Cell Cultures (DSMZ), one of the world’s largest culture collections. Most of these bacterial strains belong to three phyla: *Actinomycetota, Pseudomonadota*, and *Bacillota* (https://bacdive.dsmz.de/).

**Table 1:** Key terms and concepts in this perspective.

**Term**	**Definition**
Genome-resolved metagenomics	Metagenomic sequences are assembled and binned into genomes that can be employed in microbial community analyses
Cultivation	The laboratory process of growing microorganisms under controlled conditions, such as temperature, pH, and oxygen level, in culture media (sometimes as part of a community reduced in diversity) to promote cell division, recover viable cells, and isolate pure (axenic) cultures (when possible)
Isolation	Procedures aimed at separating a single microbial cell from a mixed community to obtain an axenic culture, involving streak plating, serial dilution, selective or differential media, and tailored incubation conditions that favour the target organism. Successful isolation yields genetically uniform populations comprising clonal descendants of a single, separated cell
Culturomics	High-throughput strategy of growth trials in which cells from microbial communities are cultivated in various types of media and under different physicochemical conditions in parallel; the resulting colonies are typically subjected to DNA sequencing for rapid assignment to taxa
Dilution-to-extinction	Microbial communities are serially diluted to reduce their diversity, ideally to a single cell
Novel species	Defined as taxa represented by genomes (typically MAGs) with <95% of average nucleotide identity (ANI) or <70 % similarity regarding digital DNA–DNA hybridization to any members of known taxa
Reverse genomics	Cultivation strategy using antibodies targeting predicted cell surface proteins inferred from genomes (or MAGs) to capture and isolate specific microorganism types
Auxotrophies	Inability of any microbe to generate specific chemical constituents needed for growth. This inability is common in environmental microbes
Perturbation	A disturbance or shift in abiotic or biotic factors, such as temperature, nutrient level, chemical exposure, or species introduction, that disrupts the structure, function, or dynamics of a microbial community
Rare biosphere	Prokaryotic species with low relative abundance or low prevalence *in situ*. For practical purposes, any species with <0.1% of relative abundance is considered rare
Flux balance analysis (FBA)	This computational and mathematical approach to analysing GEMs relies on fluxes of metabolites via network models, which is useful to predict and maximize cell growth rates
Machine learning (ML)	ML is a subfield of artificial intelligence (AI) that uses algorithms and statistical models enabling machines to build systems and to learn from data, optimizing their performance over time on a specific goal or task

Whereas genome sequencing provides essential insights into the genetic potential of prokaryotes, cultivation remains indispensable for experimentally exploring their physiology, metabolism, and ecological roles that cannot be fully inferred from sequence data alone [15–17]. This is crucial for lineages influencing key ecosystem processes, taxa with few or no cultivated representatives, and species relevant to human and environmental health or hold potential for biotechnological applications [15, 18]. For example, members of the widely distributed phyla *Patescibacteriota* and *Sysuimicrobiota* (formerly phylum CSP1-3) are among the most desirable prokaryotic lineages for cultivation because of their ecological significance and persistent uncultivability [19–21].

The urgency of safeguarding microbial diversity through cultivation efforts has intensified in the face of accelerating climate change, land degradation, and habitat fragmentation [22, 23]. These environmental pressures might threaten prokaryotic diversity, risking the loss of species before they can be identified, studied, and harnessed. Monitoring and preserving microbial diversity must therefore be discussed in future conference of the parties of the United Nations Convention on Biological Diversity (Philippe Sansonetti; https://www.pasteur.fr/en/research-journal/news/challenge-and-importance-preserving-microbial-biodiversity). Such discussions have paved the way to formally integrate microorganisms and microbiomes in global conservation and biodiversity policy scenarios, now also endorsed by the Microbial Conservation Specialist Group of the International Union for Conservation of Nature [24].

For decades, prokaryotic species have been isolated by plating microbial cells (usually from serial dilutions) on agar-based culture media. These traditional methods are often biased towards isolating aerobic, mesophilic, heterotrophic, and fast-growth species [15, 25]. The isolation of many prokaryotic taxa using traditional methods is challenging, primarily due to the disruption of microbial interactions and obligate metabolic interdependencies (e.g. vitamins produced by accompanying populations) [26, 27]. Moreover, the nutritional needs and physicochemical tolerances of yet-uncultivated prokaryotic taxa are highly specific and often unknown [15, 28].

To improve the isolation of prokaryotic species, diverse strategies have been used, such as the dilution-to-extinction method, *in situ* cultivation devices, microfluidics, cell sorting, and culturomics [15, 25, 28–30]. Examples of ingenious approaches that have yielded cultures of previously uncultivated taxa include modifications in the isolation media (e.g. using low-nutrient concentrations, growth substrate combinations, and supplementation with cAMP), cocultivation in dialysis vessels, physical enrichments via chemotaxis [31], and diffusion chamber-based approach to mimic natural environment [32, 33]. Over the past three decades, cultivation techniques have improved, enabling the isolation of a multitude of novel prokaryotic taxa. These efforts have increased the number of newly described species from a mere 100 per year (in the 1980s to 1990s) to ~1000 species per year in the last decade (https://lpsn.dsmz.de/statistics/figure/15) [34]. Yet, according to the SeqCode registry (https://registry.seqco.de), 55 phylum names and 21 673 species names (without synonym) of cultured prokaryotes are valid under the International Code of Nomenclature of Prokaryotes (as of December 2025).

In the age of metagenomics, innovative strategies (e.g. reverse genomics) have been pushing the boundaries of possibilities for isolating target prokaryotic species [35, 36]. (Meta)genome information has provided insights into the metabolic capabilities and growth requirements of specific prokaryotic taxa, guiding the development of new cultivation endeavours [16, 36–38]. However, the systematic integration of physiological profiling, genotype–phenotype inference, and genome-scale metabolic models (known as GEMs or GSMMs), derived from high-quality metagenome-assembled genomes (MAGs), has yet to be fully incorporated into targeted strategies for microbial isolation. In this regard, it has been argued that advancing cultivation efforts requires the integration of microbiome-derived data with classical microbiological methods [39].

Recently, machine learning (ML) (see definition in Table 1) has been employed to analyse the relationships between prokaryotic genotypes and phenotypes based on high-quality genomic and phenotypic data [40]. The reconstruction of ML-powered GEMs represents a substantial opportunity to improve the robustness and quality of traditional GEMs by providing more complete fluxes of metabolites and accurate environmental and metabolic context, including information about potential auxotrophies, metabolic interdependencies, substrate preferences, and niche constraints. Integration of ML, GEMs, and omics data can provide a more comprehensive link between genotype and phenotype. This joint information could offer clues for the appropriate design of cultivation strategies, including tailored culture media formulations and growth conditions [41, 42]. Although genome-based design of culture media to isolate of representatives of novel prokaryotic species is an established approach for certain species-poor microbial communities [16, 43, 44], it has not been systematically applied using MAGs representing novel taxa.

Currently, the challenges of isolation of novel or yet-unculturable taxa are technical and conceptual, and several questions must be addressed in this topic. For example: (i) How can microbial communities be reshaped in favour of the enrichment of novel taxa? (ii) How can phenotypic and physiological traits be predicted from genomes and genes with unknown functions? (iii) How can genomes (including MAGs) be systematically used to improve target isolation strategies?

### From microbiome reshaping to axenic cultures: an integrative framework

**Figure 1:**
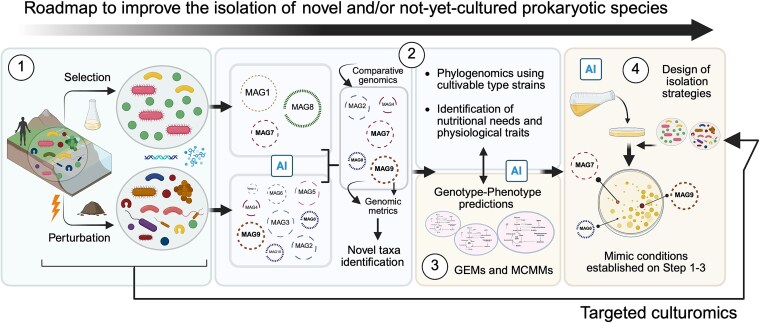
Integrative conceptual framework encompassing four steps. (1) Reshaping microbial communities via *ex situ* perturbation or targeted enrichment experiments can increase the chances of detecting hidden prokaryotic diversity by altering interspecies dynamics and triggering shifts in the microbial community. (2) Genome-resolved metagenomics and the analysis of high-quality metagenome-assembled genomes (MAGs) can provide information about the novelty of the detected prokaryotic taxa (e.g. comparative genomics) and their metabolic potential and predictive physiology. (3) Artificial intelligence (AI)–based genotype–phenotype predictions, genome-scale metabolic model (GEM) reconstruction, and microbial community-scale metabolic models (MCMMs) using MAGs (detected in Step 2) can enhance the understanding of the nutritional needs and metabolic interdependencies of species, yielding crucial information on the potential design of cultivation and isolation strategies for target prokaryotic taxa. During Steps 2 and 3, ML-based tools improve MAG reconstruction, gene function annotation in MAGs, and the accuracy of GEMs and MCMMs (see Fig. 2). (4) Based on Steps 1 to 3, targeted isolation strategies (i.e. targeted culturomics) can be established and tested, including AI-driven combinatorial synthetic culture media design. At this stage, information on physicochemical conditions applied in perturbation or enrichment culture assays is input into an AI-predictive analysis to enhance the isolation of specific taxa. BioRender.com was employed to create the figure.

This paper outlines an integrated conceptual framework, merging the controlled restructuring of microbial communities—via perturbation—with genome-resolved metagenomic analysis (including predictions of phenotypic traits and GEM development), artificial intelligence (AI)–guided culture media design, and experimental validation. This framework reimagines the standard isolation workflow by positioning microbiome perturbations or enrichment cultures, not just as an experimental condition, but as a deliberate, selective ecological filter. Perturbation experiments, enrichment culture methods, and genome-guided isolation have been refined in separate contexts, whether to probe the microbial community assembly in ecological studies [45] or to cultivate taxa with specific metabolic activities [38]. However, they have never been united in a single workflow. This perspective is not intended to ‘reinvent the wheel’; instead, it proposes combining these established approaches into a coordinated, iterative roadmap to facilitate the systematic discovery and targeted isolation of novel or yet-uncultured prokaryotic taxa. In this context, by addressing the challenges and highlighting innovation opportunities, this manuscript aims to lay the groundwork for a systematic and predictive science of prokaryotic cultivation and isolation based on improved and interpretable genome information coupled with AI-driven tools such as ML. The next sections explain and discuss the topics concerning the conceptual framework (Fig. 1).

### Improving the chance of retrieving novel taxa via microbiome reshaping

Several prokaryotic species are found in natural ecosystems in low abundance (i.e. rare biosphere) or in a dormant stage [15, 46–48]. Normally, low-abundance species are challenging to characterize via metagenome sequencing owing to the high sequencing depth required to detect them [49]. Ecological studies have proven that under changed, perturbed, or selective conditions, some rare or dormant species may become highly active, outcompeting dominant species, increasing in abundance, and contributing to community functioning [50–52]. The induced selection of these prokaryotic taxa may improve their detectability using metagenomics, increasing the chances of informing successful isolation strategies.

In this context, microbiome reshaping aims to increase the odds of detecting rare, novel, or divergent prokaryotic lineages. Two primary approaches can be employed to restructure microbial communities: (i) perturbation experiments and (ii) enrichment cultures. Commonly, these two approaches select for specific functions, but the combination of factors or dynamic shifts in physicochemical conditions could produce random changes in the structure of the original microbiomes. Phenotype or function-based enrichment can be applied in instances where no phylogenetic markers exist to track specific prokaryotic taxa in the reshaped microbial communities.

#### (i) Perturbation experiments

Acute or chronic perturbation experiments have long been employed to examine microbial dynamics, stochastic/deterministic shifts, and the resilience or resistance of microbial communities under stress conditions [53, 54]. These studies often apply biotic (e.g. introduction of invading populations) or abiotic stressors (e.g. nutrient pulses, desiccation–rewetting cycles, pH or temperature changes) directly to cells in the environmental matrix (e.g. soil or water) to mimic natural or anthropogenic disturbances [55–59]. Although microbial communities often display predictable successional patterns following a disturbance, such as transient declines in diversity followed by reassembly [45, 54], these shifts can also uncover cryptic community members previously undetectable. Such community reshuffling can enhance the likelihood of detecting novel prokaryotic taxa, especially when stressors select for traits that are underrepresented in the original microbial community, including tolerance of extreme conditions and metabolic flexibility [50, 60]. This process occurs because perturbations can alter the ecological balance or the steady state of microbial communities via competitive hierarchy disruption, resource monopolization, and niche partitioning [60, 61]. Natural ecosystems rarely settle into permanent climaxes; instead, they are dynamic and punctuated by successional turnovers over weeks, months, seasonal and diel cycles, and epicyclic swings in dominant and rare taxa [62]. Dominant taxa can successfully occupy environmental niches, suppressing the proliferation of slow-growing species via competitive exclusion. However, environmental fluctuations—here through applied perturbation—can disrupt existing microbial hierarchies by selectively disadvantaging the dominant taxa, allowing rare taxa to increase in relative abundance and exploit newly available resources or vacated niches [60, 63].

Thus, induced perturbation of microbiomes could be an approach to uncover hidden microbial diversity for elusive lineages, which can then be enriched and likely further isolated. To the best of the authors’ knowledge, very few studies have applied controlled *ex situ* perturbations to environmental samples, aiming to enrich rare and/or novel yet-uncultivated prokaryotic taxa [50], compared with the more widespread application of enrichment strategies that often rely on adding a selective substrate to favour specific metabolic traits over successive culture cycles.

#### (ii) Enrichment cultures

In enrichment cultures, a small amount of the natural sample or derived microbial cells is inoculated in selective liquid media which then favour the growth of the most adapted microorganisms able to respond to the stimulus, i.e. nutrient based or adaptative [64–66]. In these systems, strong selection pressure is imposed where the niche space and resource availability are temporarily restructured, allowing otherwise excluded taxa to expand [67]. Enrichment cultures have been instrumental in cultivating difficult-to-grow microbes, particularly those involved in specialized metabolic transformations, such as methanogenesis [27], acetogenesis [68], anoxygenic photosynthesis [69], or polysaccharide [38] and plastic [70] degradation.

These controlled systems have allowed the enrichment and cultivation of many unknown and novel prokaryotic taxa. For instance, MAGs belonging to three novel bacterial genera (i.e. *Pristimantibacillus, Andeanibacterium*, and *Mangrovimarina*) have been detected in polymer-amendment liquid enrichment cultures using tropical soil as the inoculum source [58, 71, 72]. However, enriching these novel taxa is only part of the journey; they must be brought into a pure culture to harness these organisms fully. For instance, enrichment cultures coupled with a dilution-to-extinction approach have been employed to isolate a known plastic-degrading microorganism, *Piscinibacter sakaiensis* (formerly *Ideonella sakaiensis*) [73]. A liquid enrichment culture has some advantages because it can detect species that have low growth rates, cannot grow on a solid surface, or are inhibited by agar constituents. In addition, the low microbial complexity in these systems, compared to environmental microbiomes, can facilitate the reconstruction of high-quality MAGs and accurate microbial community-scale metabolic models (MCMMs). Therefore, enrichment cultures should not be considered passive pre-cultivation steps but active ecological tools functioning as targeted, scalable perturbations that can reshape the community structure in favour of rare and/or novel prokaryotic species.

### Genome-resolved metagenomics: starting point for new cultivation endeavours

**Figure 2:**
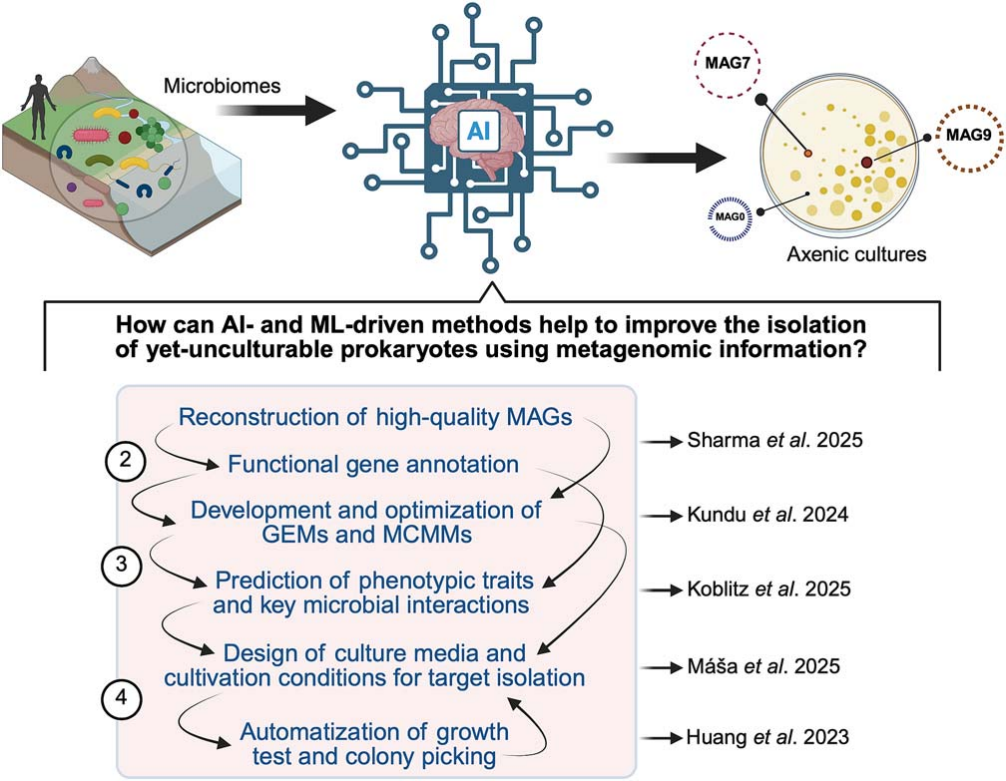
Schematic representation of the proposed steps for the target isolation of yet-uncultivable microbial species detected by genome-resolved metagenomics. Most of the processes within the pink square (i.e. from MAG reconstruction to identification of axenic cultures containing the target MAG) can be optimized by using AI-driven methods. In particular, the reconstruction and optimization (via gap filling) of genome-scale metabolic models (GEMs and MCMMs) and prediction of physiological and phenotypic traits are processes where ML-driven methods have been implemented. Arrows within the square represent input for each step and processes. BioRender.com was employed to create the figure.

Advances in metagenomics and computational tools have enabled the analysis of thousands of novel MAGs [2], offering gateways to the predominantly uncharted microbial diversity. In this regard, employing long-read DNA sequencing technologies (e.g. PacBio-HiFi and Oxford nanopore) [74, 75] and hybrid assemblies combining short- and long-read sequencing data [76] offers advantages for reconstructing high-quality (>90% completeness and <5% contamination) [77] and low-fragmented MAGs (e.g. <100 contigs) [78]. When combined with high sequencing depth and advanced bioinformatic analysis integrated into ML-based approaches [79] (Fig. 2) and supported by high-quality databases, these hybrid strategies greatly improve the detection of low-abundance taxa and characterization of intraspecies diversity based on MAGs [80, 81]. However, a considerable fraction of prokaryotic diversity with low-to-intermediate abundances still escapes genome-resolved metagenomics, affecting the characterization of microbiomes with high diversity [82, 83]. Although researchers have demonstrated a nanopore-based selective sequencing method that increases the genome coverage of rare prokaryotic taxa [84], the reconstruction of high-quality MAGs of the taxonomically broad, low-abundance species remains a challenging task. Cell-sorting techniques coupled with reconstruction of single-amplified genomes have partially solved this problem.

Curated and up-to-date databases, different genomic metrics, and comparative phylogenomic analyses are necessary to determine the uniqueness of a specific set of high-quality MAGs. Recently, thresholds and standards have been proposed for genomes if used for taxonomy, nomenclature, and delineating novel prokaryotic species [85, 86]. Phylogenomic comparisons can provide data regarding the circumscription of a novel taxon, identifying related prokaryotic species with isolated representatives (e.g. type strains). This information can be applied to guide the initial isolation attempts for targeted species by applying the cultivation conditions for their closely related isolated representatives, similar as is reported in the Known Media Database [87].

In this regard, the Bac*Dive* and Media*Dive* databases [88, 89] report comprehensive data on the physiological traits and cultivation conditions of ~100 000 prokaryotic strains and nearly all type strains. Providing information on the strain level is essential because prokaryotic physiology, metabolic functions, and ecological dynamics are conserved at different phylogenetic depths, sometimes even varying between subpopulations of the same species [90, 91]. Moreover, a significant amount of genetic variation exists within a single prokaryotic species [92–94], making cultivation efforts more challenging. For example, various strains of the same species may harbour divergent sets of biosynthetic pathways, nutrient requirements, or stress response mechanisms, affecting their ability to grow under laboratory conditions. Neglecting this intraspecies diversity can oversimplify microbial diversity, masking strain-specific functions and potentially reducing the predictive power of the GEMs employed to guide cultivation efforts.

### Functional gene annotation of MAGs as a keystone for target cultivation strategies

In addition to genome characterization using various features (e.g. ANI values, GC content, size, and number of protein-coding genes), a robust functional gene annotation is crucial to decipher the predictive metabolism of detected prokaryotic species. Currently, gene functions are primarily predicted based on their sequence similarity to previously characterized proteins deposited in databases. The use and integration of databases (e.g. KEGG, InterPro, MEROPS, Gene Ontology, CAZy, and Pfam), relaxed sequence homology thresholds (e.g. a minimum of 40% amino acid identity and a minimum of 70% sequence coverage), and genomic context [95, 96] can expand the gene annotation space, offering a comprehensive functional profiling of prokaryotic-derived genomes. Recently, a new method was developed to improve the prediction of functions in human gut microbiomes by leveraging expression data, gene proximity, sequence similarity, and a ML-based classifier system [97] (Fig. 2).

Highly relaxed protein similarity thresholds could spur errors in the assignment of potential functions. Thus, deep homology detection approaches for annotating gene catalogues are also recommended [98]. The coupling of these methods to unveil metabolic profiling and identify relevant ecological traits, such as stress tolerance [99], carbon and energy sources [100], growth rates [101], optimal growth temperatures [102], and amino acid biosynthesis pathways [103], provides useful physiological information of the target species, offering context for the design of cultivation strategies. Recently, the metabolic traits and physiological information retrieved from MAGs have offered important clues about carbon and nitrogen substrates for the isolation of the following: a fastidious crude-oil degrader from oil-contaminated seashores (*Macondimonas*; [104]), thermophilic spirochetes from a deep terrestrial subsurface biosphere (*Longinema*; [37]), a phenanthrene degrader from petroleum-contaminated soil (*Achromobacter*; [105]), an anaerobic arsenic-methylating bacterium from soil (*Paraclostridium*; [106]), and a hyperthermophilic polysaccharide degrader from hot spring sediments (*Fervidibacter*; [38]). A recent study has identified metabolic traits and pathways associated with culturability, highlighting the functional novelty found in yet-uncultured microbial species [107]. In addition, a novel genome-based computational model using amino acid frequencies was developed to predict oxygen tolerance, optimum temperature, salinity, and pH for a range of prokaryotic groups [108].

A major challenge in genome-assisted cultivation is the sequence-to-function gap because many prokaryotic genomes contain genes with unknown or poorly annotated functions that complicate the interpretation of genome-derived data (see Box 1), especially when attempting to predict the entire metabolic potential, physiology, or phenotype of a microorganism [109–112]. This limitation can impair the development and curation of GEMs and the translation of genomic information into actionable cultivation strategies. High-throughput experimental platforms (e.g. gene synthesis or CRISPR/Cas-guided gene mutations coupled with microfluidic phenotypic screening), AI-driven predictions, and accurate databases are still necessary to bridge this sequence-to-function gap [109, 110, 113]. Emerging AI-based approaches are powerful tools in protein discovery and should be widely adopted to enhance the accuracy of the current predictions of protein domain annotations. For instance, structural features extracted from amino acid sequences using prediction software, such as AlphaFold [114], could further improve gene annotation. Moreover, large language models (LLMs) have demonstrated significant superiority over state-of-the-art approaches based on protein family classification, reducing prediction errors [40, 115, 116]. Further, a flux balance analysis (FBA; Table 1) to scrutinize GEMs may serve as a framework to identify and correct candidate genes whose functions might be considered unknown during annotation but are likely to support growth, a process typically known as gap filling [117].

Box 1. **Unknown functions of MAGs**

Diverse factors can hinder the complete functional analysis of metagenomes: (i) Metagenome sequencing commonly omits a considerable fraction of the rare biosphere [111]. (ii) Gene and MAG catalogues are developed after an assembly process, and unassembled sequences are often discarded [94]. (iii) Gene annotation approaches provide vague and nonspecific functional information (e.g. transporters, transcriptional regulators, or hydrolases). In addition, many proteins (40% to 60%) derived from metagenomic gene catalogues or MAGs are categorized simply as hypothetical proteins [112]. This set of genes without functional annotation can be denominated as the ‘microbial gray matter’. This term was proposed by Dr Pedro J. Torres in a post where he aimed to clarify the concept of microbial dark matter that has been indiscriminately used in literature to refer to the unknown fraction of metagenomes and unseen not-yet-culturable microbial species (https://medium.com/@PedroJTorres_/microbial-dark-and-gray-matter-exploring-the-unknown-67fc9ac0c712). Four recent studies have computationally analysed unknown functions of MAGs found in diverse ecosystems, helping improve their functional assignments (e.g. using protein structural similarities and genomic context) and discovering new protein families [96, 97, 112, 114]. In addition, some funded research programmes (https://www.darpa.mil/research/programs/duf) and collaborative international networks (https://dark.metagenomics.eu/who.html) have been established to address this topic. There are still open questions regarding the bottlenecks of gene function annotation and how to address the unknown functions of MAGs with high-throughput experimental validations. In one example, a study of mutants derived from 32 bacterial strains growing under various conditions measured gene fitness, proposing functions for poorly annotated enzymes and transporters [118]. The science is still far from filling the sequence-to-function gap in complex microbiomes, considering the relevance of the environmental context in genotype–phenotype relationships. However, computational biology is moving forward fast, and it is predicted that AI-based tools can help prioritize protein families to validate their functions experimentally.

### MAG-based metabolic modelling and ML-aided phenotype inference

Cultivating prokaryotes by using MAG information requires translating the functional potential into testable hypotheses about physiology and growth. To this end, reconstruction of GEMs, MCMMs, and ML-based phenotype predictions can be used as complementary approaches.

#### (i) Mechanistic modelling from MAGs

An accurate reconstruction and appropriate analysis of GEMs and MCMMs can help integrate and contextualize the functional profiles of MAGs across diverse environmental scenarios. These predictive metabolic models enable a better understanding of the physiology, nutritional needs, and substrate preferences of target prokaryotic species under certain growth conditions [41, 119]. In microbial communities, MCMMs can provide information about how small molecules, such as amino acids and vitamins, in the extracellular matrix affect the cell growth of specific members, detecting potential cross-feeding events and auxotrophies [120, 121]. For example, MAG-based metabolic models have been used to predict possible metabolic exchange events and interactions in hydrothermal vents and hot spring-derived microbiomes [122, 123].

The predictive power of the resulting GEMs and MCMMs from metagenomic data is still limited due to intrinsic (e.g. low completeness of MAGs, drawbacks in gene annotation, and gaps in many metabolic pathways) [124] and extrinsic factors (e.g. lack of physicochemical context in microbial growth). Beyond improving the functional gene annotation, integrating transcriptomic, proteomic, and metabolomic data is recommended to overcome these challenges, providing insight into the overall context of the sampled microbial community, including viability, metabolism, expression levels, adaptations, and lifestyle of target-specific members [125]. This integration can be achieved by using Metabolic-Informed Neural Network that utilizes omics data to predict metabolic fluxes under different conditions [126]. Additionally, emerging ML-driven strategies contribute to refining, structuring, optimizing, and integrating data, enhancing GEMs and MCMMs via gap-filling support (e.g. AMMEDEUS and BoostGAPFILL) and improved FBA feasibility spaces [127] (Fig. 2).

#### (ii) ML for phenotype predictions

Phenotypic traits can also be inferred using ML with training sets based on the available high-quality genome and well-curated phenotypic (e.g. motility, spore formation, and salinity tolerance) and physicochemical environmental data (e.g. temperature, pH, and oxygen). These approaches can link phenotypic traits with protein families, also reducing the sequence-to-function gap in prokaryotic genomes [40]. ML-driven strategies have been used to predict optimal growth temperatures [128] and phenotypic traits [116] and to functionally classify MAGs [129] (Fig. 2). Nonetheless, these ML-based models are trained with known microbial taxa; they depend on draft genomes and partial annotation of genes and often lack the inclusion of unconventional or new metabolic pathways, and main assumptions are based on fixed environmental conditions without biological/ecological context (e.g. microbial interactions). These factors could generate low-quality predictions and false-positive associations [116, 130]; however, ML-based models still constitute a powerful pathway for phenotype predictions.

#### (iii) Integration of mechanistic models and ML-powered inferences

The above-described strategies stem from fundamentally distinct paradigms: mechanistic modelling (e.g. GEMs) seeks to elucidate biological processes through detailed physicochemical representations yet faces scalability challenges in complex microbial systems; conversely, ML-driven phenotypic inferences can predict outcomes in high dimensional contexts without requiring mechanistic insight, albeit at the cost of large, high-quality training datasets. In other words, whereas GEMs enable metabolic predictions under defined nutritional and/or genetic constraints, ML-based approaches can refine these predictions by capturing phenotypic responses to environmental factors not explicitly represented in GEMs.

Process-informed neural networks (PINNs) represent a family of hybrid frameworks that integrate prior mechanistic knowledge—expressed as differential equations or constraint-based models—into the architecture or training of neural networks. Unlike purely data-driven models, PINNs are trained not only to fit data but also to satisfy biophysical, biochemical, and ecological constraints such as mass balances, reaction stoichiometry, and thermodynamic feasibility [126, 131, 132]. These hybrid modelling frameworks can be used to predict optimal media compositions, pH, temperature, or other physicochemical parameters that maximize microbial growth. For example, closely related physics-informed variants have modelled population dynamics by embedding environmental variables directly in the loss function, achieving accurate predictions for monocultures and microbial communities [133]. Therefore, incorporating enzyme constrained metabolic models can capture proteome allocation limits and catalytic capacities, yielding more realistic predictions of substrate utilization hierarchies and aiding the rational selection of crucial nutrients for cultivation testing [134].

Taken together, combining mechanistic metabolic knowledge with ML-driven approaches within an iterative design–build–test–learn framework is poised to deliver reliable genotype-to-phenotype inferences from MAGs and to improve targeted culturomics. In practice, hybrid GEMs/MCMMs, accounting for the influence of environmental factors, might predict cultivation conditions for target prokaryotic taxa, reducing the resources, time, and labour required for extensive testing. These hybrid modelling paradigms have the potential to provide mechanistic information that can be sensitive to extrinsic variables, making more accurate predictions [135, 136].

### Transforming genomic clues into an *ad hoc* isolation strategy

Reshaping microbiomes may provide improved access to hidden prokaryotic diversity, but a significant bottleneck remains because numerous species continue to escape isolation in axenic culture. Traditional isolation methods may be unsuitable for some prokaryotic taxa if they fail to replicate the specific conditions of their natural habitats, including the presence of key growth factors [25]. With this goal, various culture media have been designed to re-create the nutritional milieu occurring in the ecosystem (e.g. soil-, seawater-, or host-extract agar) [137, 138]. Efforts in designing new solid or liquid synthetic culture media are vital to improve the isolation of target prokaryotic species [37, 139]. The nutrient conditions found in a hot spring have recently been employed as the basis for the design of semi-solid culture media to target the isolation of chemolithotrophic iron-oxidizing bacteria [140]. Although agar-agar has been used for ~150 years as a gelling agent for solid media, gellan gum has helped isolate a representative of the lineage WOR-3 from hydrothermal vents [141]. This factor and the bias associated with solid culture media must be considered in any targeted isolation strategy. Moreover, buffered culture media might restrict the growth of certain species types and certain buffers have long been known to inhibit bacterial growth; thus, adapting or even avoiding buffer solutions could be a critical choice for cultivating target taxa [142, 143].

In the future, AI-based models could help to conduct a proper and optimized synthetic culture media design [42, 130]. Although these approaches have not been extensively evaluated for the target cultivation of prokaryotic species, they could reduce the scope and rapidly screen thousands of predictive nutrient formulations, increasing the likelihood of a successful isolation [144, 145]. Recently, an AI-based tool was developed to learn the amino acid requirements for two oral streptococci [146]. In addition, rule-based classifiers and LLMs have been used to predict culture media preferences using microbial traits beyond genomic features or phylogeny [147]. These pioneering studies could accelerate the AI-guided design of culture media (Fig. 2). As mentioned, the metabolic data retrieved from MAG-derived genes, MAG-based metabolic models, and MMCMs can be applied (Fig. 1, see Steps 2 and 3; Fig. 2) to facilitate identifying unknown essential nutritional requirements, potential auxotrophies, cofactor dependencies, and environmental tolerances of specific microbes. This process can be fuelled by using complementary tools, such as DRAM [148], gapseq, and CarveMe [120], which perform functional annotation and GEM reconstruction. Such diverse data can guide the design of AI-assisted culture media and adjust cultivation parameters, such as carbon and nitrogen sources, vitamins, metals, pH, redox potential, temperature, or oxygen levels, which specifically cater to the physiological needs of target taxa [37, 41]. Active learning and Bayesian optimization frameworks can iteratively refine media composition using experimental growth feedback, accelerating the identification of optimized culture conditions [149, 150].

In this context, isolation of target prokaryotic species can be tested via a specific culturomic approach, where cultures can be analysed using Polymerase Chain Reaction (PCR) with specific probes [151] or using mass spectrometry [152] to detect the target prokaryotic taxa (Fig. 1, see Step 4). Recently, culturomic-based metagenomics has enriched specific prokaryotic species in human gut microbiomes [30] and has captured some of the unknown microbial diversity in desert soil [153]. Current culturomic strategies and growth media optimization efforts often rely on empirical methods. Thus, genome-guided culturomics transform a trial-and-error process into a hypothesis-driven one, improving the odds of isolating target species that have not yet been cultured. However, this *ad hoc* strategy must be supported by sophisticated technologies, heavy infrastructure, new methods, complementary approaches (see Box 2), and special cultivation workflows, especially for anaerobic microorganisms [125, 154].

Box 2. **Complementary innovations to improve the isolation of prokaryotes**

Identifying biosynthetic gaps or substrate dependencies from MAGs offers information on the requirements for cocultivation, conditioned media, or externally supplemented growth factors. Thus, culture media targeting specific microorganisms can be combined with other methods to avoid disrupting complex networks and metabolic interactions commonly occurring in the ecosystems. In cocultivation, syntrophic partners provide essential metabolites to enhance the growth of auxotrophic taxa [155]. For example, an approach called the sandwich agar plate was developed, aiming to coculture various populations throughout the isolation [156]. For liquid cultures, cocultivation technology is well established, using vessels in which partners are separated by dialysis membranes [157]. An anaerobic bioreactor-mediated enrichment allows the cocultivation of a pure syntrophic community, recovering a target archaeal taxon (*Promethearchaeum syntrophicum*; [158]). In the future, single-cell and CRISPR technology [159] could improve the isolation of specific target species from complex microbial communities. For example, taxon-specific probes derived from target MAGs can enable targeted cell sorting for cultivation and isolation in a selective culture media designed for its growth [160, 161]. Another interesting approach includes labelling cells, detection based on the physiological state or response to environmental changes, and isolation mediated by cell-sorting techniques [162]. In addition, AI-based models coupled with robotics can improve the detection of microcolonies (Fig. 2) that cannot be detected by the human eye, increasing the chance of isolation of the target species [163, 164].

## Concluding remarks

By integrating various approaches, methods, models, and tools, the proposed framework represents a multidisciplinary gateway to discovering and isolating novel or yet-uncultivated target prokaryotic taxa. In this context, perturbation and enrichment culture experiments offer powerful means to reshape microbial communities under controlled conditions, promoting the potential of discovering taxa that are otherwise difficult to detect in natural systems. Advancing these wet-laboratory strategies can permit access to previously elusive microbial lineages, enhance opportunities for cultivation, and broaden the knowledge of microbial diversity and ecosystem functioning.

Although all efforts to characterize genomes, genome-based models, and MMCMs seem complex, they can generate comprehensive and useful information regarding the metabolic and physiological profiles of target prokaryotic species, offering a foundation to refine culture media formulations and design innovative isolation strategies. However, due to the drawbacks and barriers in MAG reconstruction and gene function annotation, gaps in metabolic pathways, and limited representation in databases, this approach is still far from a systematic application. Experimentally annotating the vast number of uncharacterized genes is an expensive, time-consuming, and daunting task and a significant hurdle facing genome-driven cultivation. A better understanding of protein functions, physiological features, metabolic pathways, and nutritional needs based on MAGs can provide the basis to isolate thousands of unknown prokaryotes. These obstacles are becoming increasingly surmountable with the convergence of advanced bioinformatics and emerging AI-based tools to assist in functional annotation [165] and synthetic media designs, defining a new era of genome-informed microbiology.

Emerging AI methods (e.g. AI-assisted gene annotation, AI-based predictions of physiological traits, AI-improved GEMs, and AI-guided media formulation) can generate vast data and new hypotheses to test the cultivation of prokaryotes. For example, an announced collaboration between the Align Foundation and American Type Culture Collection can train and validate AI-based models that predict microbial physiology from genetic information, helping to predict cultivation conditions for numerous species (align-announces-new-collaboration-with-atcc-to-bring-ai-ready-microbial-datasets-to-life).

Fostering synergy between microbiome scientists, computational and system biologists, and microbiologists would facilitate and accelerate the high-throughput isolation of novel or yet-uncultivated prokaryotic taxa, uncovering ecological roles and biotechnological potential. Finally, we pose provocative and forward-looking questions: (i) Are researchers sequencing a prokaryotic world that is increasingly challenging to cultivate, isolate, or fully understand? (ii) Without the availability of microbes as pure cultures, can researchers understand microbial life in depth or harness it for real-world applications? These questions are aimed at inspiring discussion, collaboration, and innovation in the scientific community.

## Data Availability

No datasets were generated or analysed during the current study.
